# Maxillofacial injuries in severely injured patients

**DOI:** 10.1186/s13032-015-0025-2

**Published:** 2015-06-09

**Authors:** Max J. Scheyerer, Robert Döring, Nina Fuchs, Philipp Metzler, Kai Sprengel, Clement M. L. Werner, Hans-Peter Simmen, Klaus Grätz, Guido A. Wanner

**Affiliations:** Department of Surgery, Division of Trauma Surgery, University Hospital Zurich, Raemistrasse 100, 8091 Zürich, Switzerland; Division of Cranio-Maxillo-Facial and Oral Surgery, University Hospital Zurich, Zurich, Switzerland

**Keywords:** Maxillofacial injury, Trauma, Head injury

## Abstract

**Background:**

A significant proportion of patients admitted to hospital with multiple traumas exhibit facial injuries. The aim of this study is to evaluate the incidence and cause of facial injuries in severely injured patients and to examine the role of plastic and maxillofacial surgeons in treatment of this patient collective.

**Methods:**

A total of 67 patients, who were assigned to our trauma room with maxillofacial injuries between January 2009 and December 2010, were enrolled in the present study and evaluated.

**Results:**

The majority of the patients were male (82 %) with a mean age of 44 years. The predominant mechanism of injury was fall from lower levels (<5 m) and occurred in 25 (37 %) cases. The median ISS was 25, with intracranial bleeding found as the most common concomitant injury in 48 cases (72 %). Thirty-one patients (46 %) required interdisciplinary management in the trauma room; maxillofacial surgeons were involved in 27 cases. A total of 35 (52 %) patients were treated surgically, 7 in emergency surgery, thereof.

**Conclusion:**

Maxillofacial injuries are often associated with a risk of other serious concomitant injuries, in particular traumatic brain injuries. Even though emergency operations are only necessary in rare cases, diagnosis and treatment of such concomitant injuries have the potential to be overlooked or delayed in severely injured patients.

## Background

Facial injuries, in particular soft tissue injuries and fractures of the facial bones, are frequently occurring as a result of motor vehicle crashes, falls, violent assaults, and crashes during recreational activities such as bicycling and skiing [1, 2].

**Fig. 1:**
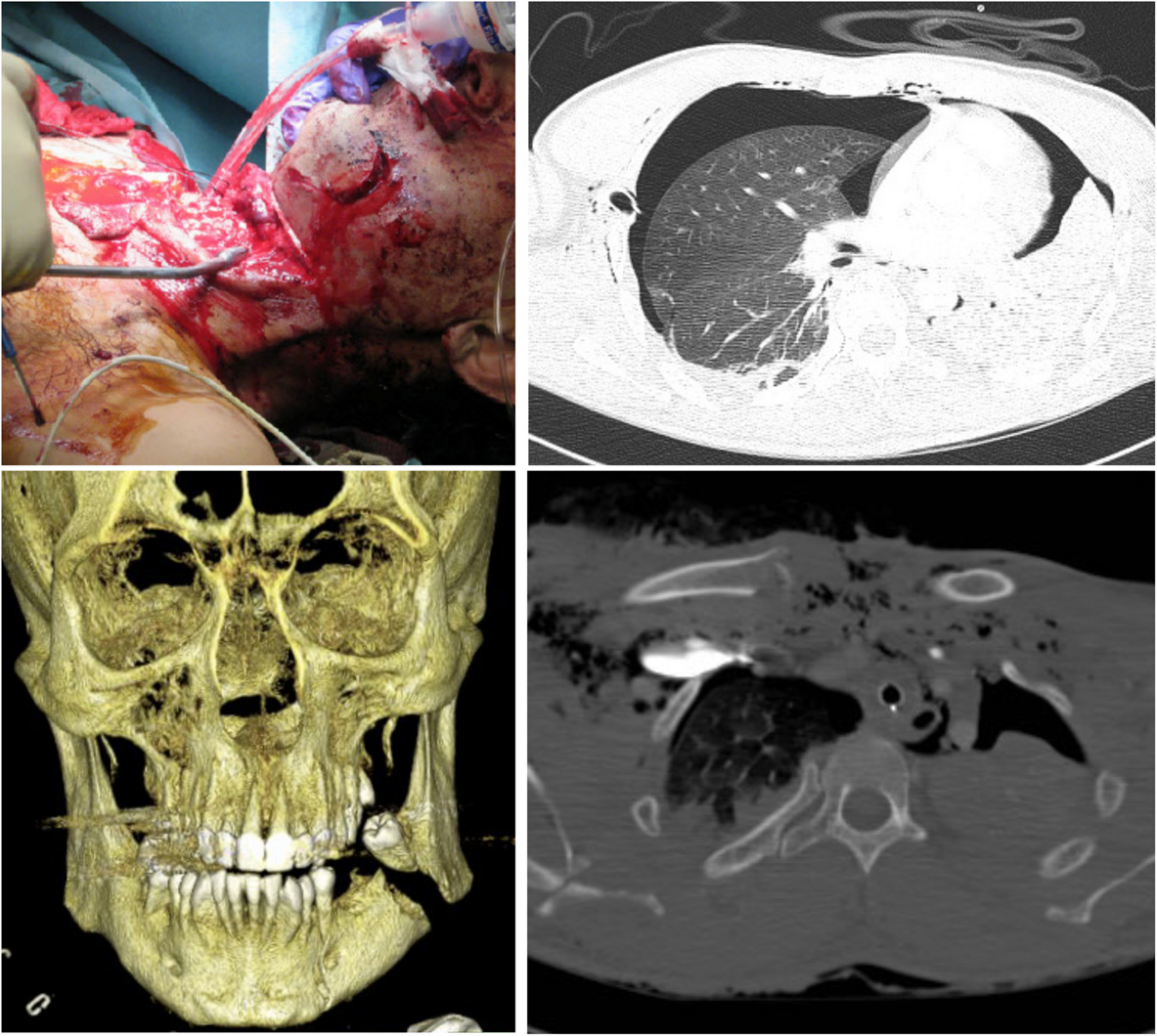
A 37-years-old patient who was hit by a pipe through the car windscreen. As a result, the patient suffered, beside a fracture of the mandibular, severe cervical (destruction of the larynx, cervical spine fractures, thyroid gland and esophagus lesion), and chest trauma (serial rib fracture, lung contusion, clavicles fracture, soft tissue emphysema). The initial treatment of the mandibular fracture within the trauma room included provisional bony stabilization. Definitive treatment was carried out after stabilization of the patient three days after trauma (Fig. 2)

**Fig. 2:**
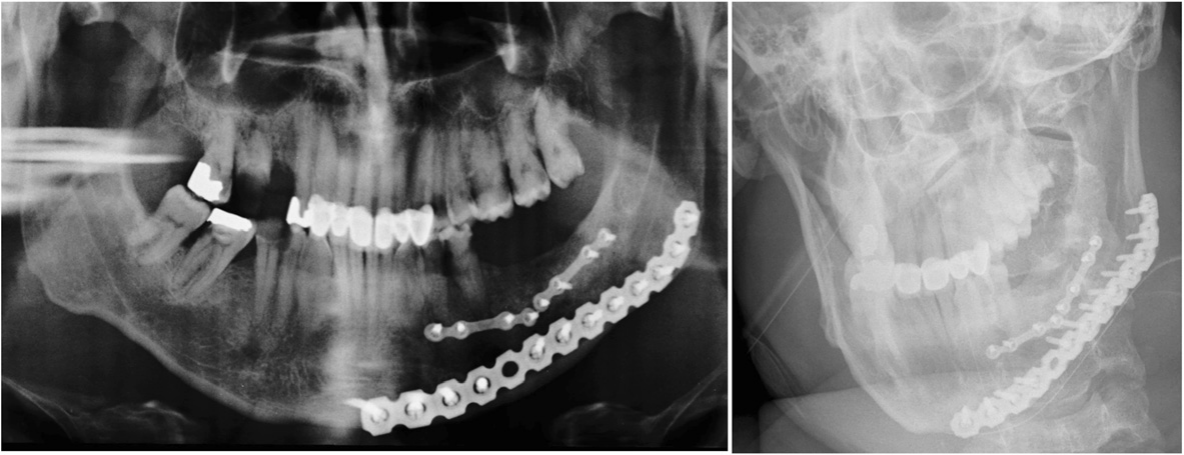
Definitive treatment of the abovementioned mandibular fracture of a 37-years-old patient three days later

While they occasionally occur as isolated lesions, they are more commonly associated with other serious injuries [3]. Previous studies demonstrate that the rate of concomitant head injuries in cases of facial fracture is as high as 50- 80 %- depending on the location of the fracture [4]. While intracranial injuries occur most often in cases of fractures of the bones of the upper face and maxilla, they are less frequently associated with lesions of the mandible [4–6]. Besides involvement of the head, other concomitant injuries include the cervical spine and other body parts (Figs. 1, 2) [3, 7, 8].

**Fig. 3:**
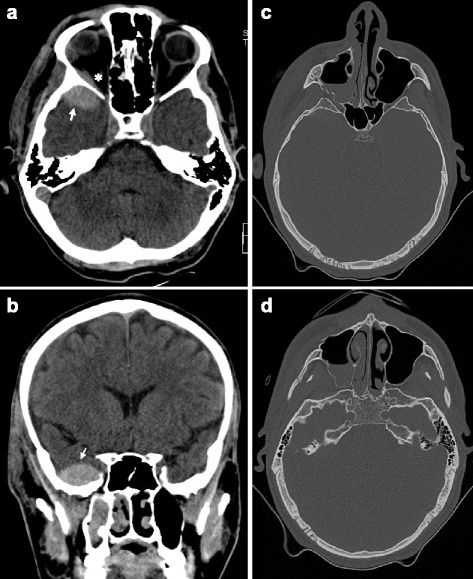
A 24-years-old patient with epidural hematoma and fracture of the orbital floor, lateral orbital wall, fracture of the sinus maxillaris, and zygoma fracture. A haematoma compression of the optical nerve resulted that was decompressed by a maxillofacial surgeon in collaboration with an ophthalmologists in the trauma room

To reduce morbidity and mortality, early recognition of severe head trauma and concomitant injuries remains an important part of the initial assessment and treatment plan of severely injured patients. Understanding the cause, severity, and distribution of facial trauma and the concomitant injuries can help in the optimization of the initial clinical treatment and definition of the right time to involve oral surgeon. In this context, it has recently become more commonly recognized that patients with sustained multiple injuries benefit from an early multidisciplinary management in a specialized trauma center (Fig. 3) [3].

The majority of the existing studies on facial injuries focused on a single type of concomitant injury like brain or cervical spine [9–11]. Others broach the issue of concomitant injuries with a particular type of facial fracture [12, 13].

The aim of the present study is to present a comprehensive view on facial and associated injuries in severely injured patients. Additionally, the role of plastic and maxillofacial surgeons in the management of this collective of patients is to be evaluated.

## Methods

Severely injured patients with facial fractures and who were admitted to the trauma division were included in the present retrospective study. The observation period was two years. All trauma patients aged 16 years and older with an injury severity score (ISS) of ≥ 17, a threshold commonly used to define severely injured patients, were routinely entered into the trauma database. Patients with an age lower than 16 were directly admitted to the children’s hospital and were therefore not included in this study.

All patients underwent a whole body computed tomography performed with contrast at admission (Somatom Definition, Siemens, Munich, Germany; 128-slice dual source CT; 120 kV, 210 mAs, slice thickness 3 mm). Fracture classification was conducted by a radiologist, neuro-radiologist, trauma surgeon and in case of involvement by a plastic or cranio-maxillo-facial surgeon.

The following parameters were collected and examined retrospectively: gender, age at time of injury, circumstances regarding the mechanism of injury, abbreviated injury scale (AIS) as well as ISS, and concomitant injuries and treatment thereof. Concomitant injuries were defined as any major injury outside the facial region and were identified according to body region and severity. Furthermore, we analyzed vital parameters, laboratory parameters, length of hospital stay, length of intensive care unit (ICU) stay, overall mortality rates, and finally cause of death.

With regard to facial injury, the observed fractures were divided into isolated fractures and complex fractures involving the respective bone.

Before the final statistical analysis, the data was pre-checked in order to ensure the necessary data quality. Mean values for interval variables and median values for ordinal variables were calculated. Correlations between ordinal variables were tested using Spearman’s Rho. For correlations between ordinal and interval variables the Kruskal-Wallis-Test was performed. Differences in AIS face between particular groups were analyzed using the Pearson Chi-Square Test and confidence intervals.

A probability value of < 0.05 was considered statistically significant. All statistical analysis was performed using IBM SPSS Statistics software (Version 21.0; IBM Corp., Armonk, NY).

Prior to data acquisition, the ethics committee approved of the study.

## Results

**Fig. 4:**
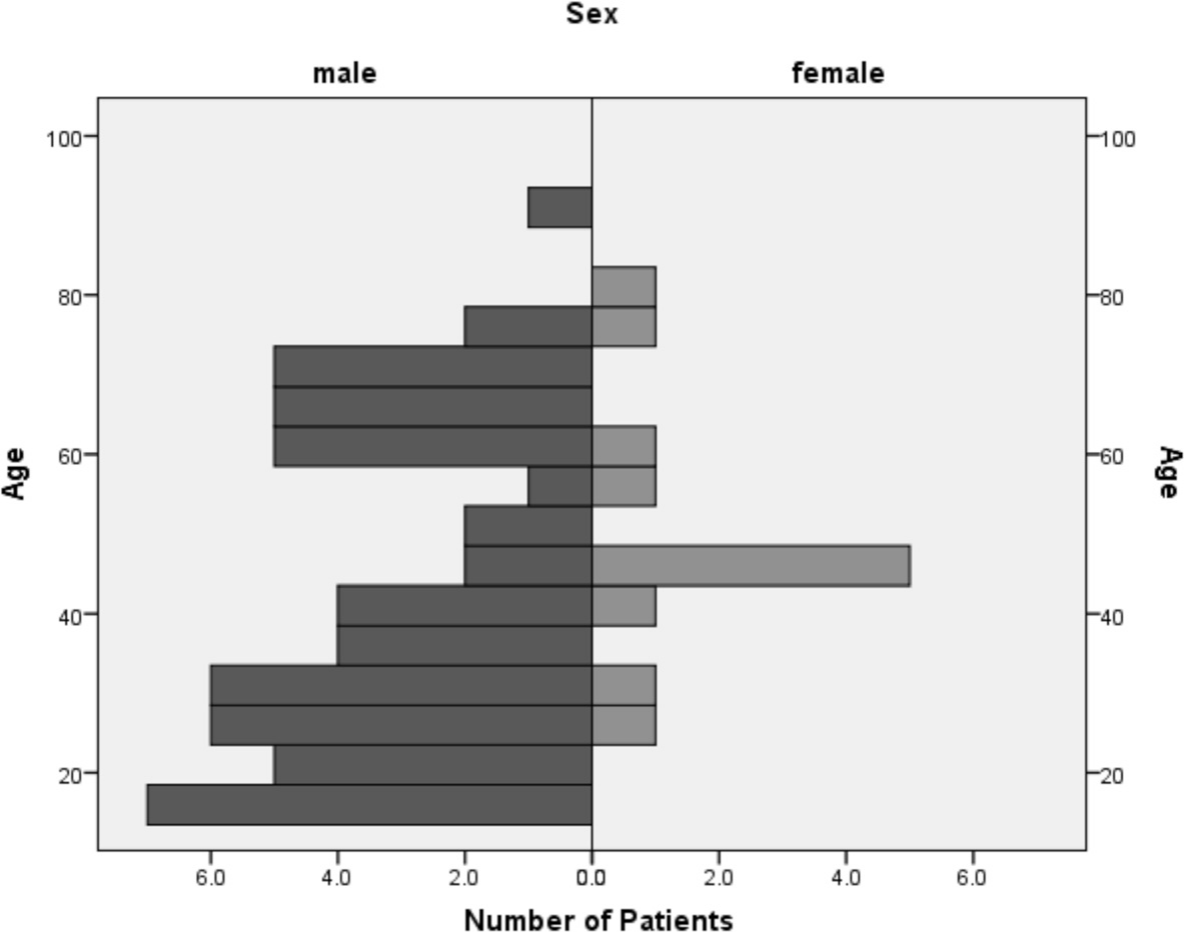
Distribution of age and sex at the time of injury

Within the study period, 487 severely injured patients with an ISS of 17 or greater were admitted to our emergency room. Fourteen per cent had concomitant maxillofacial injuries (n = 67). Fifty-five (82 %) of these patients were male and 12 (18 %) were female, resulting in a ratio of almost 5:1 (Fig. 4). The age ranged from 16 to 91 years with a mean age of 44.

The most common cause of injury were falls (37 %, n = 25), in most cases from a level below 5 meters (24 %, n = 16), followed by motor vehicle collisions (MVC) (21 %, n = 14) and bicycle accidents (19 %, n = 13). Almost all patients involved in MVCs were driving the vehicles themselves (93 %, n = 13). Other causes of injury were pedestrian-car-accidents and assaults. Penetrating injuries appeared in 6 cases (9 %).

A large fraction of the patients suffering facial trauma was injured during late afternoon and evening, between 16:00 and 24:00 (31 %, n = 21). Almost half of the injuries occurred on weekends including Fridays (48 %, n = 32). Regarding the monthly distribution, a higher number of injuries occurred during the summer months (June to August, 33 %, n = 22).

**Fig. 5:**
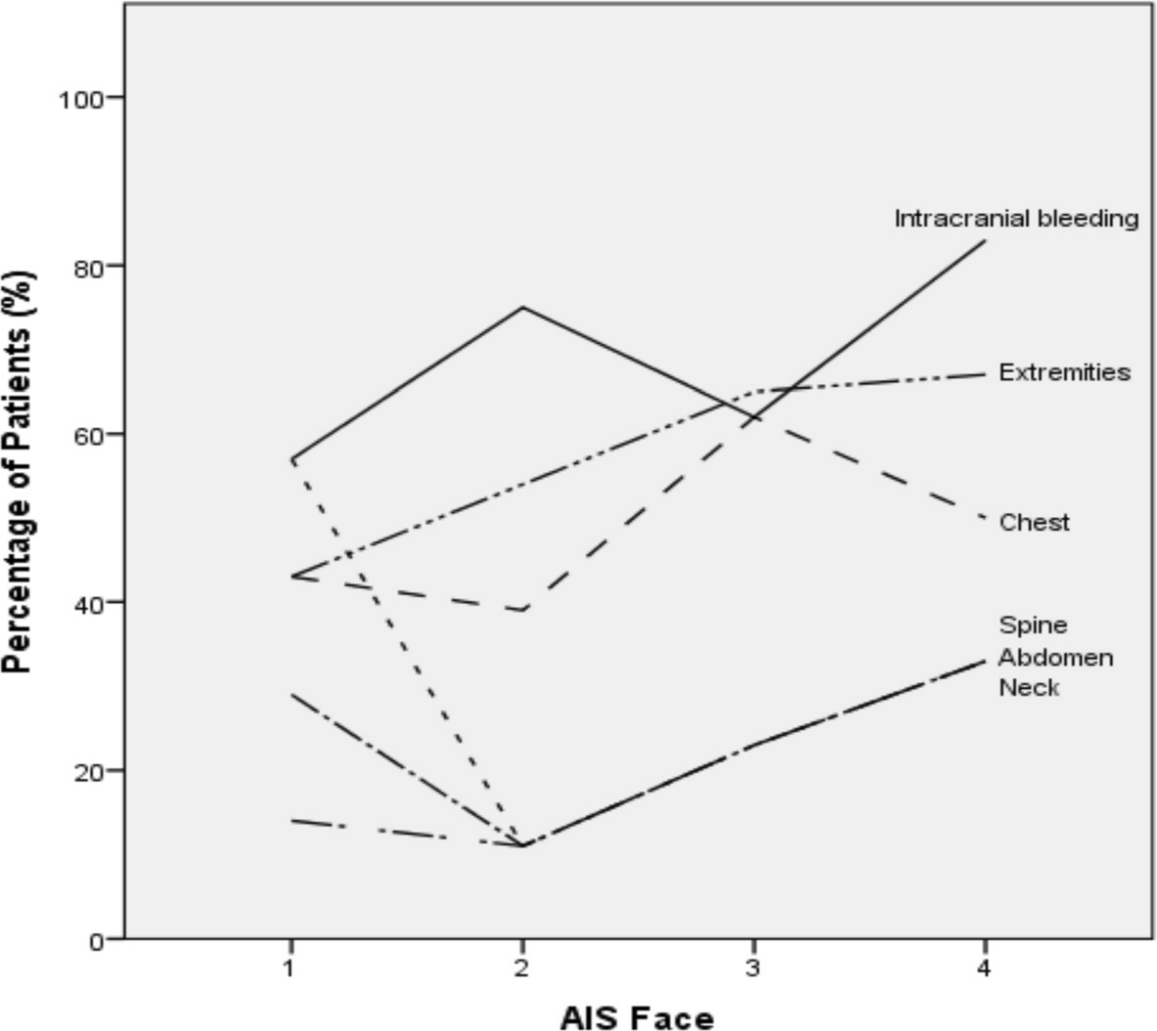
Associated Injuries in relation to the AIS Face

A median ISS of 25 (range 17-57) was identified. The most common injuries associated with facial fractures were intracranial bleeding (72 %, n = 48), injuries of extremities (58 %, n = 39), and the chest (49 %, n = 33) (Table 1). More severe facial injuries were associated with a higher scale of concomitant injuries (Fig. 5).

**Table 1:** Associated injuries of 67 patients. Injuries are defined as fracture, luxation, major contusion or wound and lesions of intestinal organs

Associated Injury	n (%)
Intracranial Bleeding	48 (72 %)
epidural	19 (28 %)
subdural	24 (36 %)
subarachnoidal	12 (18 %)
parenchymal	13 (19 %)
Neck	12 (18 %)
Chest	33 (49 %)
Abdomen	13 (19 %)
Spine	15 (23 %)
Extremities	39 (58 %)

**Fig. 6:**
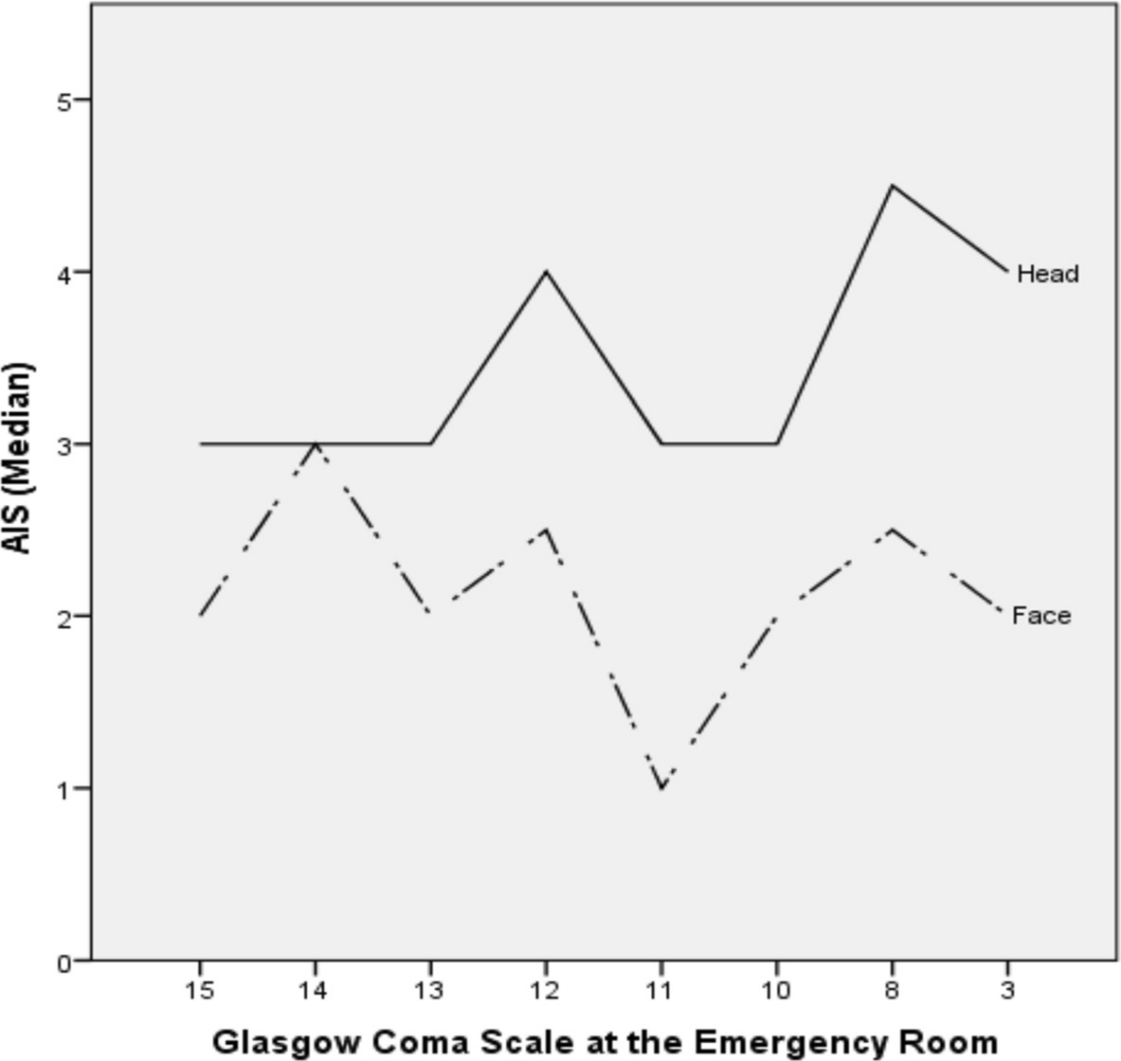
Correlation between Glasgow Coma Scale at the Emergency Room and AIS Face and Head for 63 patients (4 missing). There was no correlation between GCS and AIS Face (Spearman’s rho, p = 0.43) but there was a correlation between GCS and AIS Head (Spearman’s rho, p = 0.02)

Regarding parameters of clinical examination, no correlation between the Glasgow Coma Scale, registered prehospitally as well as at the Emergency Room, and the AIS Face (Spearman’s rho prehosp.: p = 0,94; 17 cases missing; ER.: p = 0,43; 4 cases missing) was identified. Hence, correlation between the GCS in ER and the AIS Face could be observed (Spearman’s rho p = 0.02; 4 cases missing) (Fig. 6). Compared to self-breathing patients, patients who arrived intubated did not have a more severe facial fracture pattern (Pearson Chi-Square, p = 0.4).

A positive blood alcohol level at admission was found in 11 cases (Mean level: 29.4 mmol/l, range 9.7 – 51.1 mmol/l). However, data about 23 patients were missing due to omitted blood tests. No correlation between a higher blood alcohol level and the AIS Face was observed (Kruskal-Wallis, p = 0.4).

The most common overall facial bone fracture was orbital (78 %, n = 52), followed by maxillary (70 %, n = 47) and zygomatic (55 %, n = 37) fractures. The most common isolated fracture was of the mandible (6 %, n = 9) (Table 2). Total maxillary fractures contained Lefort-fractures, which were found in 16 cases (24 %).

**Table 2:** Facial fracture pattern for 67 patients

Fractures	n (%)
*Orbital Region*	
Total	52 (78 %)
Isolated	3 (5 %)
*Maxilla*	
Total	47 (70 %)
Isolated	0 (0 %)
*Zygoma*	
Total	37 (55 %)
Isolated	1 (2 %)
*Nasal Bones*	
Total	30 (45 %)
Isolated	3 (5 %)
*Sphenoid bone*	
Total	21 (32 %)
Isolated	0 (0 %)
*Mandible*	
Total	18 (27 %)
Isolated	6 (9 %)
*Frontal bone*	
Total	15 (22 %)
Isolated	0 (0 %)
*Teeth*	
Total	12 (18 %)
Isolated	2 (3 %)
*Petrosal bone*	
Total	11 (17 %)
Isolated	2 (3 %)
*Temporomandibular joint*	
Total	7 (11 %)
Isolated	4 (6 %)
*LeFort-Fractures*	
LeFort I	6 (9 %)
LeFort II	1 (2 %)
LeFort III	9 (13 %)

All patients included in this study got an interdisciplinary treatment consisting of trauma surgeons and, according to fracture pattern, plastic surgeons or cranio-maxillo-facial (CMF) surgeons. In nearly half of the cases, a specialist in one of the two areas was directly present at admission at the emergency room (46 %, n = 31; plastic surgeon: 6 %, n = 4; CMF surgeon: 40 %, n = 27).

Half of the patients were treated operatively (52 %, n = 35). In 7 cases (10 %), emergency surgery was necessary directly after admission. The other patients underwent surgery after an average hospital stay of 7 days (range 2-22). A higher AIS Face is associated with a higher rate of operative treatment (Spearman’s rho, p = 0,001).

The mean in length of hospital stay was 20.3 days (range 3-60). A higher AIS Face was significantly related to a longer hospital stay (Spearman’s rho, p = 0.02).

## Discussion

Severely injured patients often exhibit injuries in the maxillofacial region, ranging from small lacerations to multiple and life-threatening fractures of the facial bones. In the literature the incidence of these concomitant facial injuries in multiple injured patients range between 15 and 22 % [3, 14]. The result in the present investigation is in line with these findings. However, comparison between epidemiologic investigations is difficult due to differences in study population, geographic region, and socioeconomic status. Further, the time of the year can influence the results as demonstrated in the present collective. In our investigation, injuries occured mainly in June and August. This observation might be explained by the circumstance that the data was collected at a level-one trauma centre located in an urban setting and most of the injuries were caused by motorcycle and bicycle accidents. In contrast, increasing incidences during the winter can be observed in regions located nearby the mountains due to higher rates of skiing accidents [2].

Although many studies have investigated the epidemiology of maxillofacial injures, most of them focus on a single type of concomitant injury like brain or cervical spine [9–11], have emphasized on concomitant injuries with a particular type of facial fracture [12, 13], or have investigated facial injuries in response to trauma mechanism. Although only few examinations exist, that pertain to the incidence of maxillofacial injuries in a general population of severely injured patients [1, 3], the data of most of the underlying investigations was collected twenty years ago and safety precautions have since significantly improved. Therefore, the aim of the present study is to give a comprehensive overview of maxillofacial injuries in a general population of severely injured patients in central Europe at the present time.

Nevertheless, our results support previous investigations- even those from other regions of the world- which have stated that road-traffic accidents are the most common causes of maxillofacial injuries [15]. In our study, 52 % of the patients were involved in road accidents either as a pedestrian, driver of a motor vehicle or bicycle. Beside this, falls were the second most common etiology, whereby most of them occurred from a height of under five meters. As a cause of injury, assaults only play a minor role in the present study population compared to previous investigations [16]. Reason might be that the inclusion criteria for this analysis are restricted to severely injured patients with an ISS ≥ 17. A correlation with the level of alcohol intoxication could not be demonstrated due to incomplete information. However, in more than 16 % of the cases, a positive finding on blood alcohol test was given. Due to missing data of 23 patients (34 %) the rate will most likely even be higher. Besides the obvious relation between alcohol abuse and an increased risk of injury, no correlation could be verified between alcohol intoxication and severity of head injury. While other investigations postulated a significantly higher number of severe head and facial injuries in the presence of alcohol [17], the present result confirmed our previous findings [18].

In the investigation at hand, most of the injuries occurred between 16:00 and 20:00. This observation might be a consequence of the abovementioned large proportion of road accidents. Normally, traffic volume is highest at this time of the day, as people tend to be on their way home from work. Due to assaults and alcohol-associated injuries, a second injury peak could be observed between 22:00 and 2:00 with increasing incidences at weekends.

Young men were the predominantly affected group of patients [19–21]. The observed gender distribution of almost 5:1 is in a line with the observation by Van Hoof *et al.*, which was made thirty-five years ago.

As mentioned before, 14 % of all severely injured patients suffered maxillofacial fractures. Hereby, mandibular fractures were the most common isolated injury comparable to previous investigations where isolated fractures occur in 33 % to 76 % of all injuries [22]. Differences in percentages might be explained by the fact that other investigations did not exclusively include severely injured patients.

Orbital (78 %) and maxillary (70 %) fractures were the most common facial bone fractures in our patient collective, indicating that they occur more commonly in complex trauma than any other facial bone fracture. Total maxillary fractures in the form of LeFort fractures occured in one fourth of the patient collective, whereby LeFort III fractures are the most common type.

Due to the high level of force required for this type of injury, the high number of concomitant intracranial lesions is not surprising. Previous investigations revealed that LeFort and frontal sinus fractures are the strongest predictors of cranial injuries in maxillofacial trauma patients. In case of frontal sinus fracture, the risk of head injury is 84-times higher; in LeFort II fractures 27-times [23, 24]. Due to the relatively small patient collective, we did not analyse statistical relation between fracture type and risk of intracranial injury like previous investigations did before [25].

Our investigation confirm that intracranial bleeding is the most common concomitant injury in patients with maxillofacial trauma [1, 7, 14]. It was observed in 74 % of our patient collective. Most of the affected patients have suffered severe midfacial fractures. Concussions were not considered in our investigation, which might explain differences to other studies suggesting brain injuries in up to 98 % [1].

Another aim of this investigation was to work out a correlation between AIS face and presence of concomitant injuries, prehospital condition, and various outcome parameters. The observed relationship between more severely maxillofacial injuries and higher AIS head values indicates that facial fractures are a good sign that the patient has suffered a level of potentially brain- damaging energy. Due to this observation, and the high incidence of concomitant brain injuries in maxillofacial trauma, it is justifiable to assume potential brain injuries for all patients with any kind of facial injury until proven otherwise. Beside the abovementioned correlation between brain and facial injuries, a similar correlation was made according to chest, abdomen, spine and extremity injuries. Hence, lesions of the cervical spine must be suspected in every patient with maxillofacial fracture, too. In the present study, almost half of all spine injuries occured in this area.

By inference, one would expect a clear correlation between low prehospital and emergency room GCS and higher AIS face. However, this correlation was not statistically significant. Therefore, it is very important to maintain a high level of suspicion for intracranial lesions in all patients with maxillofacial trauma, even those with no obvious signs and symptoms of brain injury.

To come to a conclusion, our results show that more severe face injuries are associated with higher rates of concomitant injuries. This relation underlines the necessity to do further diagnostic workup in cases with severe facial trauma to make sure that no injury is overlooked.

Further, the wide distribution of injuries implicates the importance of close collaboration between trauma-, maxillofacial- and neurosurgeons. Indications for immediate maxillofacial intervention are tracheotomy, if no orotracheal intubation is possible; nasal packing, balloon tamponade or direct compression in severe bleedings and hemodynamic unstable patients. In cases of instable midface fracture, primary fracture reduction and preliminary fixation can be necessary (fixation of mandibular fractures and if necessary mandibulomaxillary fixation) to achieve a stable support in order to avoid permanent facial deformity and eases management and convalescence of the patients. In cases of partial or complete visual loss due to direct or indirect optic nerve trauma, e.g. with bone fragments affecting the optic nerve, retrobulbar hematoma or emphysema immediate decompression is indicated.

In our investigation, half of the maxillofacial injuries required operative intervention, while emergency surgery was not required in most cases. However, the presence of a maxillofacial surgeon seems to be inevitable, especially for airway control and hemostasis. The latter was necessary in seven cases.

**Fig 7:**
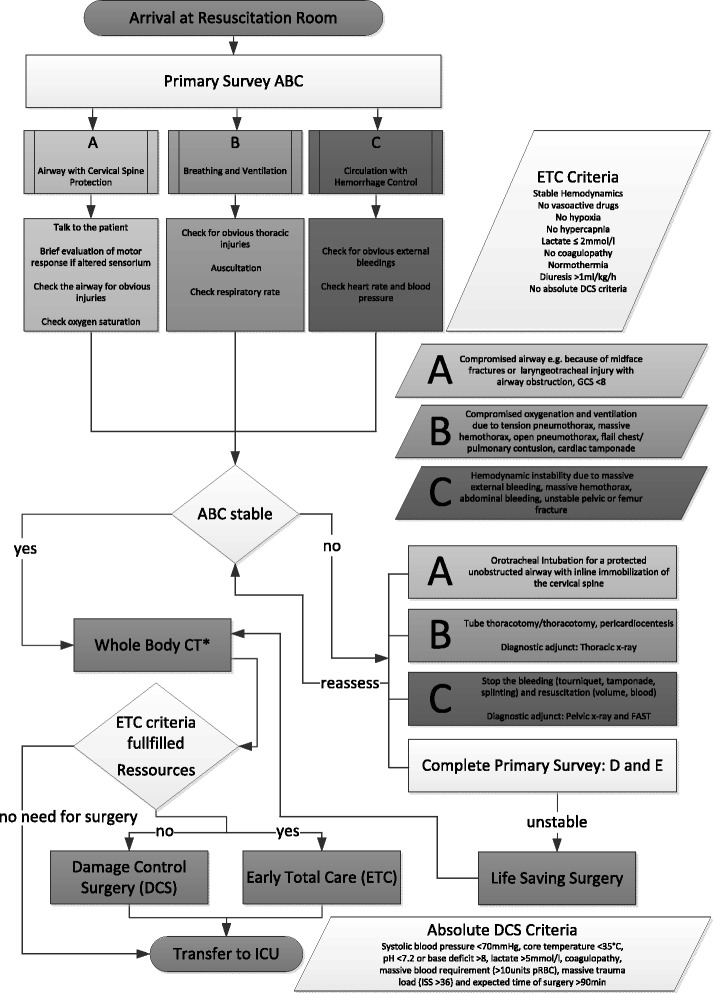
ATLS based algorithm of a diagnostic and therapeutic work-up in severely injured patients. Immediate involvement of a maxillofacial surgeon is necessary in accordance with the ATLS algorithm: A: midface fracture with obstructed airway; C: Severe nasal and/or oral bleeding. Instable midface fracture with severe bleeding; D: Partial or complete visual loss due to direct or indirect optic nerve trauma, retrobulbar hematoma or emphysema. (*Special Requirements of the Whole Body CT: Need for standard protocol, which should include the midface and mandible, if injuries of the midface are obvious. In case of no other life threatening injuries, if the standard protocol does not include vascular sequences of the head and neck, this should be done in hemodynamic stable patients direct after the primary scan is completed)

In order to ensure all these points, a careful diagnostic and therapeutic work-up is necessary as shown in the algorithm in Fig. 7. Further a multidisciplinary team should be available to perform primary evaluation and treatment of the patient.

We acknowledge several limitations of the present study. First, due to the retrospective nature of this design, we were depended on complete and accurate patient medical charts to evaluate the physical condition on admission. Although data collection was done in a routine setting by trained personnel of the Trauma centre, we cannot ensure the completeness of the data with certainty. With regard to concomitant injuries, CT scans were assessed without knowledge of previous findings, and afterwards compared with the previous diagnosis. Therefore, completeness can be ensured. Second, the study was undertaken at a single designated trauma centre. This might cause selection bias and, thus limiting the external validity of the findings. Third, the low number of fractures hinders interpretation of the data. Accordingly, no regression modelling was performed to evaluate statistical relations between the different injuries.

## Conclusion

In conclusion, our study shows a high rate of concomitant injuries with facial trauma. In particular, the high number of accompanying intracranial lesions emphasizes the need to screen all trauma patients (with facial fracture) for brain injuries, irrespective of obvious signs and symptoms. Therefore, the routine use of a head as well as full-body CT scan for all severely injured patients is recommended to ensure that no concomitant injury is overlooked. Nevertheless, the need for immediate maxillofacial surgery was low, although it seems necessary that treatment of severely injured patients should be limited to major trauma centres, wherein close collaboration between trauma-, neuro-, and maxillofacial surgeons can be ensured.
